# Wandering Bullet Embolization in Suicidal Near-Contact Gunshot Wound

**DOI:** 10.7759/cureus.26607

**Published:** 2022-07-06

**Authors:** Abdurrab A Kasim, Faisal M Alzubaidi, Yahya M Zakari, Naif A Aljohani, Raed M Alobaidaan, Raghad M Assiri, Samah F Ibrahim

**Affiliations:** 1 Forensic Medicine, Forensic Medicine Center, Riyadh, SAU; 2 Forensic Medicine, King Fahad Security College, Riyadh, SAU; 3 Forensic Medicine, College of Medicine, Imam Mohammad Ibn Saud Islamic University, Riyadh, SAU; 4 Department of Clinical Sciences, College of Medicine, Princess Nourah bint Abdulrahman University, Riyadh, SAU; 5 Forensic Medicine, College of Medicine, Cairo University, Cairo, EGY

**Keywords:** autopsy, embolization, chest, bullet, suicide, forensic science

## Abstract

Suicidal firearm injuries with bullet embolization following wandering bullet path are infrequent findings where the penetrated bullet could not be detected in the expected location. If this condition exists, one entrance wound will be present without an exit wound. Through necro-radiographs and postmortem autopsy, forensic experts can determine the nonlinear trajectory of the bullet. To understand the internal bullet path properly, forensic experts should interpret the medicolegal investigation results in the context of tissue and ballistics factors. Various medical specialties, including forensic experts, should be aware of the possibility of the nonlinear bullet trajectory and the possibility of bullet embolization in distant sites in order to save lives and/or interpret the collected evidence to support the justice in such uncommon incident.

## Introduction

Suicidal firearm injury resulted in 67,500 deaths worldwide in 2016 that represented 30.5% of firearm deaths [1]. The bullet path in firearm deaths is determined by postmortem radiology and dissection [2]. Generally, the bullet leaves the body through an exit opposite to its entry. However, the bullet path can be altered after bouncing off an internal structure and it can be retained in the body [3]. In this study, we have discussed the unusual wandering bullet path in a loose-contact suicidal handgun wound.

## Case presentation

**Figure 1:**
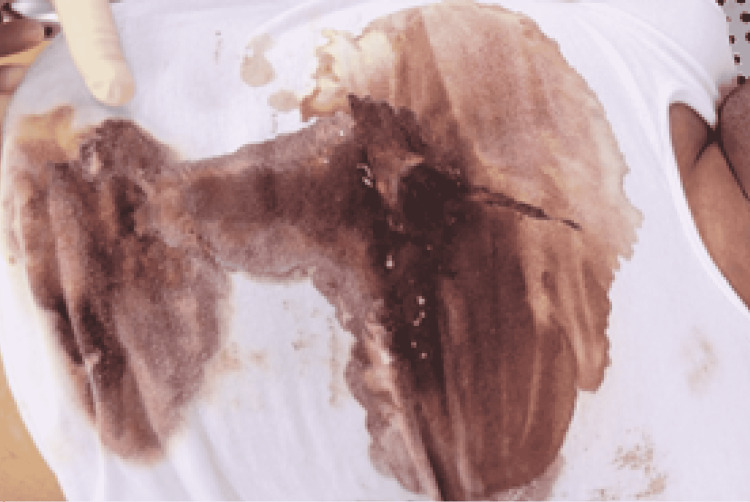
T-shirt showed a 1-cm circular hole surrounded by a 3-cm circular blackening and was soiled with blood

Police reported that a 25-year-old man was found dead in his bedroom that was locked from the inside, and a revolver handgun was detected beside him with no evidence of foul play. A single firearm wound was also noted in the middle of his chest. Police transferred him to Forensic Medicine Center for postmortem forensic evaluation. On external examination, a third-decade male wearing a white T-shirt and underpants was found to have a firearm injury in the middle of his chest without an exit wound. The front of his t-shirt was soiled with blood and revealed a 1-cm circular hole surrounded by a 3-cm circular blackening, which they determined to be soot (Figure 1).

**Figure 2:**
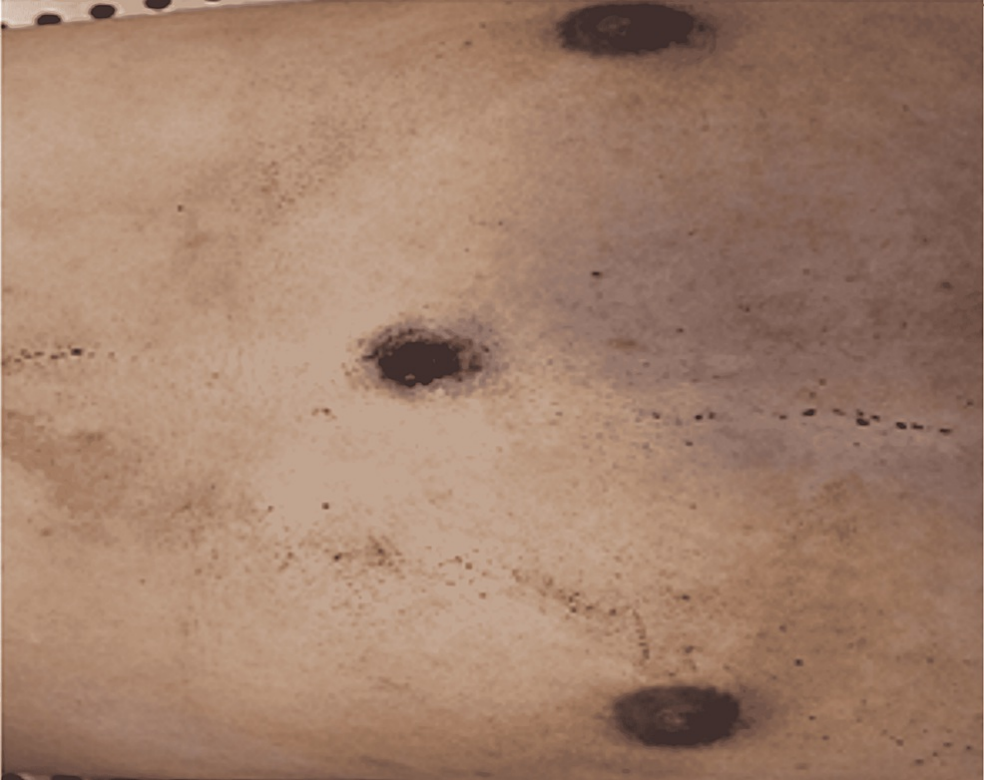
The midline of the lower part of the chest had a 0.5-cm circular defect with abraded burnt edges surrounded by a 2-cm circular blackening

**Figure 3:**
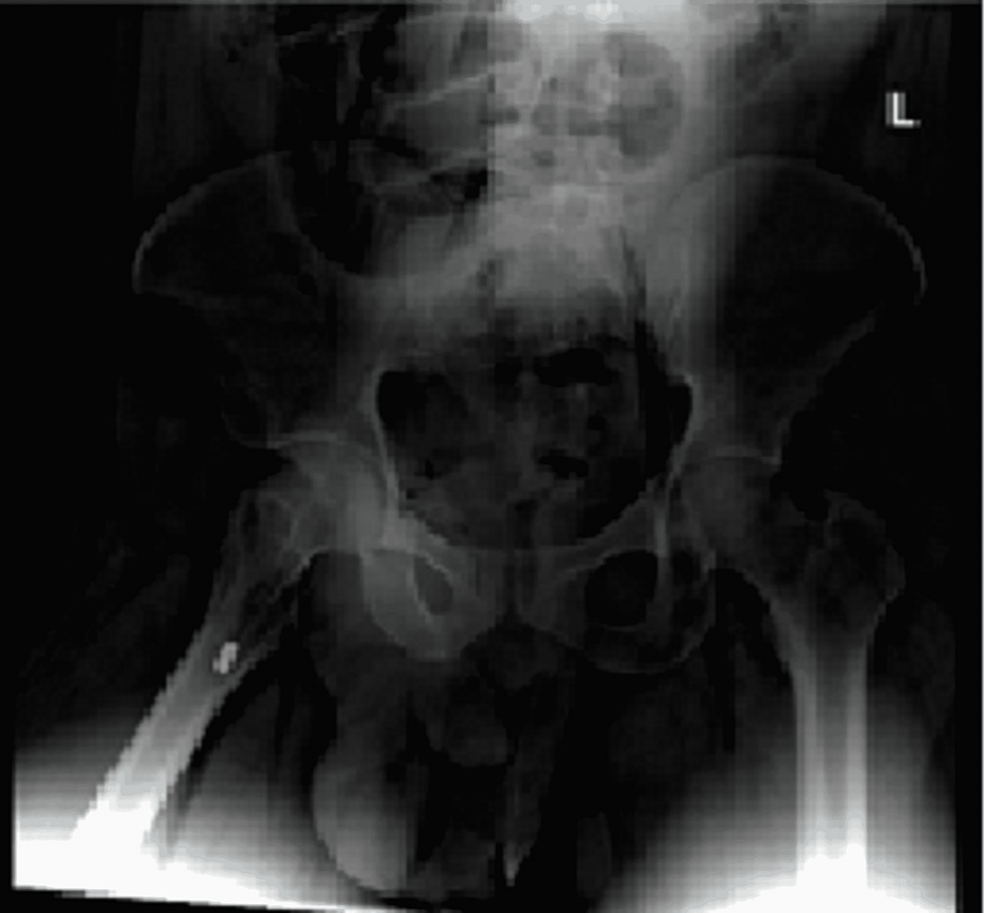
Forensic postmortem pelvic and upper part of lower limbs radiograph L: left.

A circular defect with abraded burnt edges surrounded by a circular blackening was found on the midline of the sternal region, 47 cm below the vertex, which had a 0.5 cm diameter (Figure 2). Forensic radiology failed to locate the bullet in the chest, the expected area, and located it in the medial upper area of the right thigh (Figure 3).

**Figure 4:**
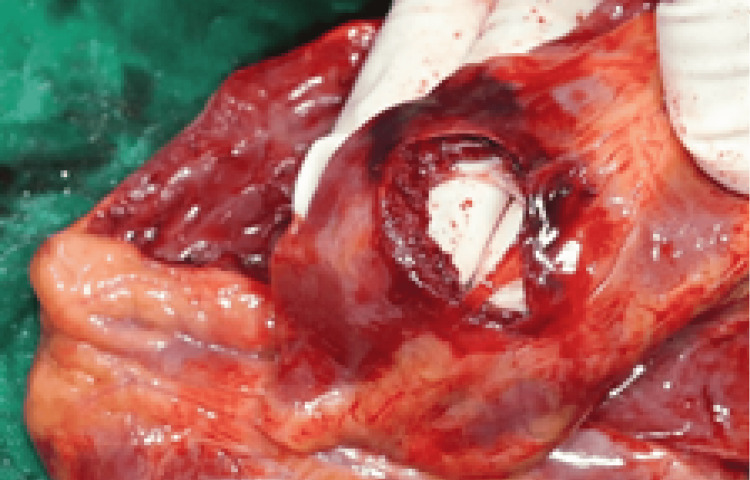
Macroscopic image showed the handgun wound in the heart

**Figure 5:**
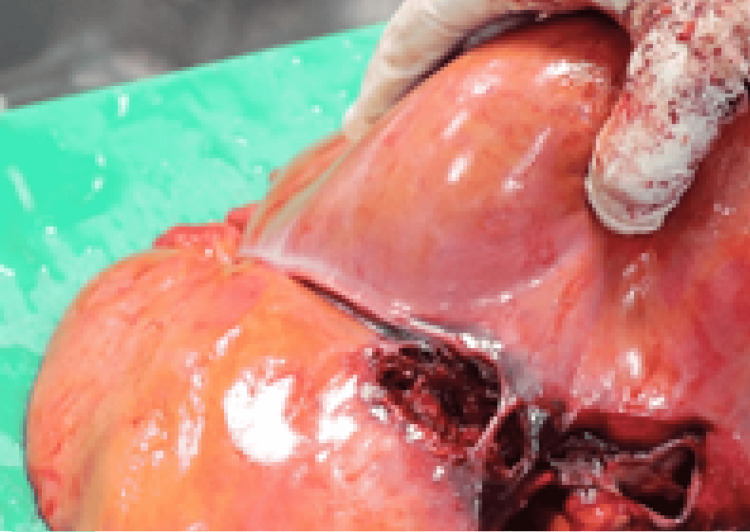
Macroscopic image showed the firearm wound in the liver

**Figure 6:**
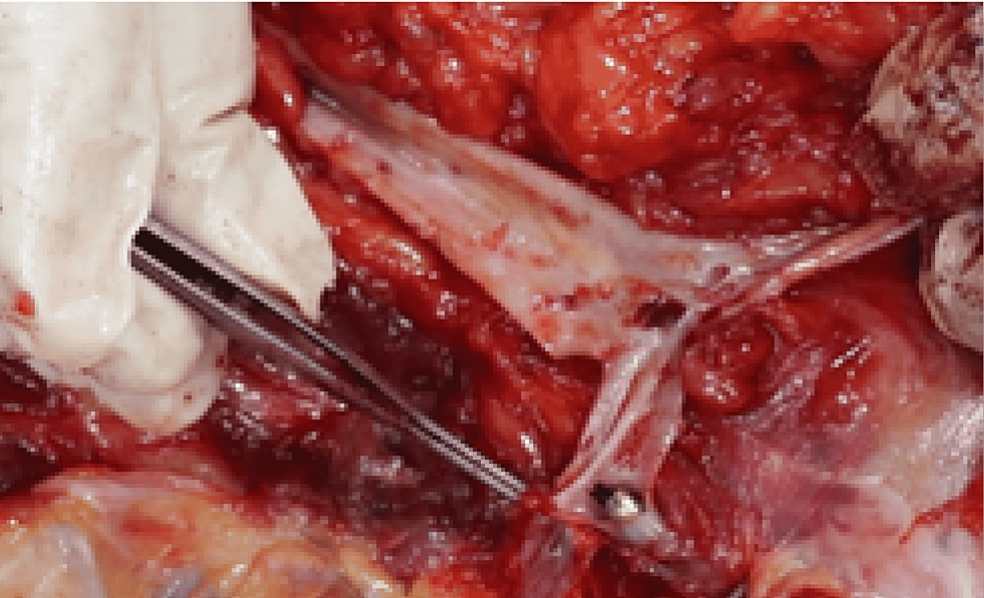
The macroscopic image of the embolized bullet within right femoral artery

Postmortem dissection was performed to detect the bullet path and mechanism of death. It revealed that the bullet in the chest was directed backward, entered the chest cavity by passing through the xiphisternal joint without resulting in any fracture, and passed through the pericardium and the right ventricular inferior wall, inflicting a tear of 2.5 cm x 1 cm (Figure 4). As the bullet trajectory was deflected downward through the diaphragm, the bullet caused a 2.5 cm x 1.5 cm tear at a posterior superior area of the left hepatic lobe (Figure 5). Then, it penetrated the abdominal aorta and followed the blood flow to be lodged in the right femoral artery (Figure 6). During postmortem examination, precordial (400 ml), intraperitoneal (700 ml), and retroperitoneal (around aorta) hemorrhages were detected. No other injuries or pathological evidence was observed.

Postmortem forensic chemical analysis revealed that alcohol level in the victim’s blood, liver, and renal specimens was 0.0 mg/ml, 0.11 mg/100 g, and 19 mg/100 g, respectively. In this case, hypovolemic shock caused by cardiac, hepatic, and abdominal aortic injuries resulting from a thoracic handgun wound was recognized as the mode of death.

## Discussion

In the present case, postmortem examination and dissection revealed that the bullet initially entered the chest and passed through the right ventricle followed by the liver and abdominal aorta and then passively migrated down to the right femoral artery. Postmortem dissection should be conducted in firearm deaths with bullet migration and embolization to analyze the firearm wound to support medicolegal investigations in detecting the bullet path and mechanism of death [4,5].

The bullet penetrates the body in a straight path, unless it is deflected or interfaced by bony hard structures where it can be lodged or be migrated. The incidence of bullet migration in penetrating firearm injuries is 0.3% according to Zahid et al. [6]. This migration can be influenced by muscular movement, increased pressure in thoracic and abdominal regions, vascular blood flow, gravity, and body movements [6,7].

In the current case, passing through sternum and facing dense cardiac muscle could help in deflecting the bullet from its straight path, thus having a tangential hit of a right ventricular wall into the liver. The amount of the inflicted damage that is seen in the heart and liver can be determined by tissue type, in addition to ballistic factors, including the bullet’s mass, caliber, shape, constitution, velocity, trajectory, and spin motion [8].

The anatomy of the liver, tissue consistency, and surrounding structure might direct the bullet in the present case to penetrate the abdominal aorta, as it lies slightly to the left of the midline of the body and inferior vena cava that has a close relation to the liver [9]. However, the bullet with low kinetic energy could only traverse one wall of the aorta, migrated with the blood flow, lodged, and embolized in the right femoral artery.

Arterial embolization is the most common type of vascular bullet embolization where bullets generally enter the arterial system through the aorta. Moreover, low-velocity small bullets with low kinetic energy are not able to penetrate further and pass with the flow of blood [4]. Primary or secondary substance use disorders, especially alcohol, are more frequently seen in completed suicides [10]. Nevertheless, the present case did not show blood alcohol levels, and the low detected concentrations in the liver and kidney might be related to postmortem production rather than antemortem consumption due to negative case history, atypical fluid and tissue distribution of ethanol, the low concentration of ethanol present, and the failure of alcohol detection in vitreous humor sample [11].

## Conclusions

This case shared most of the suicidal firearm characteristics, such as gender, firearm use, and single entry point. However, it shared infrequent characteristics, including the bullet path through the heart, where it was deflected to the liver, with successive aorta penetration and right femoral artery embolization. Detecting bullet path and the cause of death need multifactorial medicolegal investigations, including thorough postmortem dissection. Complementary medicolegal investigations collect evidence that establishes the mode and the manner of death.

## References

[1] Naghavi M, Marczak LB, Kutz M (2018). Global mortality from firearms, 1990-2016. JAMA.

[2] Hejna P, Šafr M, Kramář R, Kučerová ŠP, Zátopková L, Sairaj RT, Janík M (2022). Reversed configuration of the muzzle imprint mark in a pistol contact entrance wound mimicking a non-suicidal act. Forensic Sci Int.

[3] Caushi F, Skenduli I, Mezini A, Rulli F (2021). Extraction of a bullet floating in the pulmonary artery after a gunshot wound. J Int Med Res.

[4] Chao J, Barnard J, deJong JL, Prahlow JA (2018). A case series of anterograde and retrograde vascular projectile embolization. Acad Forensic Pathol.

[5] Picoli FF, Mundim-Picoli MB, Alves AM, Silva MA, Franco A, Silva RF (2020). Suicidal tandem bullets to the heart with subsequent embolization: a case report. Forensic Sci Med Pathol.

[6] Zahid I, Rahim Khan HA, Irfan O (2016). Retrograde bullet migration from inferior vena cava into right common iliac vein following gunshot: a case report. J Pak Med Assoc.

[7] Schroeder ME, Pryor HI 2nd, Chun AK, Rahbar R, Arora S, Vaziri K (2011). Retrograde migration and endovascular retrieval of a venous bullet embolus. J Vasc Surg.

[8] Hanna TN, Shuaib W, Han T, Mehta A, Khosa F (2015). Firearms, bullets, and wound ballistics: an imaging primer. Injury.

[9] Tucker WD, Shrestha R, Burns B (2021). Anatomy, abdomen and pelvis, inferior vena cava. StatPearls [Internet].

[10] Brådvik L (2018). Suicide risk and mental disorders. Int J Environ Res Public Health.

[11] O'Neal CL, Poklis A (1996). Postmortem production of ethanol and factors that influence interpretation: a critical review. Am J Forensic Med Pathol.

